# Improved cosmetic and functional outcomes with V-shaped mini flap and dermofat graft combination for revision of cleft lip scars: A retrospective observational study

**DOI:** 10.1097/MD.0000000000050101

**Published:** 2026-08-07

**Authors:** Mehmet Fatih Akkoc, Volkan Ozel, Mehmet Bayram, Semra Bulbuloglu, Ibrahim Ugur Budak, Berk Tahhan, Ali Haydar Cinbas, Ismail Yildiz

**Affiliations:** a Department of Plastic Reconstructive and Aesthetic Surgery, Faculty of Medicine, Dicle University, Diyarbakir, Turkey; b Department of Plastic Reconstructive and Aesthetic Surgery, Şanliurfa Training and Research Hospital, Sanliurfa, Turkey; c Division of Surgical Nursing, Department of Nursing, Faculty of Health Sciences, Istanbul Aydin University, Istanbul, Turkey; d Department of Midwifery, Faculty of Health Sciences, Istanbul Aydin University, Istanbul, Turkey; e Department of Biostatistics and Medical Informatics, Faculty of Medicine, Dicle University, Diyarbakir, Turkey.

**Keywords:** advancement-rotation V-shaped mini flap, alveolar cleft, cleft lip, dermofat graft, reconstruction

## Abstract

Cleft lip is a craniofacial anomaly, a developmental defect originating in the embryonic period. Cleft lip repair is most often performed between 3 and 6 months after birth. The scar that develops after repair is aesthetically and functionally improved through revision using various grafting techniques. The V-shaped mini-flap method is frequently used for the closure of mini-microform cleft lips. The aim of this study was to present the cosmetic and functional results. This was a retrospective observational study conducted from June 1, 2018 to June 30, 2024 including all patients who underwent secondary cleft lip revision. All patients had previously undergone primary cleft lip reconstruction, and all underwent revision for secondary cleft lip repair. A V-shaped mini flap was used to correct vermilion deviation, and a dermofat graft was used to prevent collapse. Some patients underwent simultaneous alveolar bone grafting. Numerical data were calculated by frequency analysis. The sample consisted of 30 patients who underwent secondary cleft lip revision. After primary cleft lip repair, whistling deformity occurred in 23.3% of the patients, vermilion irregularity in 26.7%, depression in 23.3%, pigmentation in 73.3%, and abnormal lip movements in 56.7%. Cleft lip was bilateral in 20% of patients and 40% had a history of concomitant cleft palate. Intraoperatively, 60% of the patients underwent V-shaped mini flap and 40% underwent V-shaped mini flap + alveolar bone graft. After secondary revision, hematoma developed in 6.7%, graft loss in 20%, and donor site morbidity in 13.4%, wound dehiscence, seroma in 10%, cyst formation in 6.7%, 10% of the patients were reoperated on. Secondary revision with a V-shaped mini flap or V-shaped mini flap + alveolar bone graft appears to be somewhat advantageous in correcting cosmetic and functional defects that arise after primary cleft lip repair, as it is associated with few manageable complications and may require a third cleft lip repair.

## 1. Introduction

The craniofacial anomaly, which is common in humans and occurs due to the lack of formation of upper lip connections during embryonic development, is called cleft lip.^[1,2]^ This congenital developmental defect manifests itself as the separation of the upper lip into 2 halves, forming an acute angle. In complete cleft lip, the 3-layered defect involving mucosa, skin and muscle continues along the upper lip and extends to the base of the nose.^[3]^ Mostly cleft lip repair is performed between 3 and 6 months after birth.^[1,4]^ Cleft lip repair performed with various surgical techniques almost always results in postoperative scarring.^[5]^ Scar development after cleft lip repair may lead to psychosocial problems as well as impaired eating and speech functions.

Various grafting techniques have been reported to improve functional and aesthetic outcomes in cleft lip revision.^[6]^ Adipose tissue can reduce inflammation and increase tissue repair.^[6,7]^ Schwaiger et al compared the efficacy of silicone implants, dermal grafts and Abbe flaps in lip revision and reported that using dermofat grafts was highly effective in preventing scarring of the lower lip as well as increasing the vermilion appearance of the upper lip.^[8]^ Dermofat graft is a very successful method to give the lip a natural appearance and volume with fat infiltration.^[9-11]^ Resnick et al examined the change in labial contour after dermis-fat graft placement and reported that it significantly improved the contour of the median tubercle. In the same study, significant increases in labial surface area and labial height were observed after grafting.^[12]^ In cleft lip, revision is required at the vermilion border, whiteroll and orbicularis oris muscle. In a previous case series of 6 cases with microform cleft lip closed with the V-shaped mini flap method, the mean age at surgery was 13.3 months (6–45 months), and after removal of the whiteroll and vermilion border, revision of the nasal cavity floor depression and orbicularis oris muscle was performed, and no significant scar was found after 1 year.^[13]^

In cleft lip cases, scar development is at the level of lip height, and the size of the scar is usually not affected by the surgical closure technique. The V-shaped mini flap method is frequently used for closure of mini-microform cleft lip. After mini/microform cleft lip repair, nasolabial revision is often required at advanced ages, but mini-microform cleft lip repair does not require revision.^[14,15]^ Even if the V-shaped mini flap method is effective in mini-microform cleft lip, its combined use with another method may be more advantageous because it may be insufficient in the revision of mini/microform. The aim of this study is to present the cosmetic and functional results with a V-shaped mini flap and dermofat graft combination for revision of cleft lip scars.

## 2. Materials and method

### 2.1. Research design and participants

The type of this study is a retrospective observational study. The results of patients who underwent cleft lip revision using the V-shaped mini flap method in combination with dermofat graft between June 1, 2018 and June 30, 2024 in a research and application hospital in the Southeastern Anatolia region of Turkey were evaluated. All eligible patients who underwent cleft lip revision during the study period were included (n = 30). All surgeries were performed under the supervision of the same surgeon. After ethics committee approval and written informed consent were obtained, the data were retrospectively extracted from the electronic patient records and recorded on a data recording form developed specifically for this study. Patients with cleft lip revision were followed up every day in the first 7 days postoperatively; patients without complications were discharged, and patients were checked on the 14th and 28th days and at the end of the sixth month, and the results were recorded. During each follow-up visit, the incision site and the surrounding area of each patient were photographed and added to the electronic patient record. Subsequently, information, documents, and photographs were retrospectively extracted from the aforementioned records and compiled, transforming them into the data for this study. There were no patients missing information, documents, and photographs; no patients were excluded; all available patients were included in the sample.

### 2.2. Data collection methods and tools

A data recording form prepared by the researchers with the support of the literature was used for data collection. The data recording form included questions about age, gender, cleft lip characteristics, other diseases, smoking history in adults, and short- and long-term complications after surgery.

### 2.3. Surgical technique

All patients who underwent alveolar bone grafting in the same session were operated on under general anesthesia, and only patients who underwent the V-shaped mini flap were operated on under local anesthesia. A surgical technique similar to the technique mentioned by Lida and Watanabe (2017) in their study was adopted.^[13]^ The apex of the scar tissue was determined. Two points on the vermilion border of the scar tissue were determined, and excision was planned in an inverted V-shape by combining with the vertex. Two points on the border of the scar were drawn in a V-shape for excision from the lip mucosa. A 3 mm triangular flap was designed at the vermilion border and the red line border on the lateral side. A 3 mm incision was made medially at the vermilion border and the red line border. Scar tissue was excised after local anesthetic infiltration. Mucosa excision was performed. The orbicularis oris muscle was dissected, and scarred muscle tissue was excised.

The inguinal region was selected as the donor area for the dermofat graft. A fusiform drawing was made in the inguinal crest according to the size of the defect. After local anesthesia infiltration, the marked 6 × 1 cm donor area was desepithelialized. The dermofat graft was harvested. The dermofat graft was planned to match the deficiency of the recipient area. Suturing was started by bringing the flaps on the red line end to end. The mucosa was sutured, and then the orbicularis oris muscle was sutured with overlap. The dermofat graft was placed over the orbicularis oris muscle with the dermis side up. The skin was sutured. In patients who were to undergo alveolar bone grafting with cleft lip scar revision, alveolar cleft repair and then cleft lip scar revision were performed in the same session under general anesthesia.

After local anesthesia infiltration, the medial and lateral mucoperiosteal flaps designed at the cleft margin were lifted. To ensure adequate mobilization of the posterior flap, the flap was released with a back-cut incision towards the buccal sulcus without damaging the alveolar bone surrounding the roots of the teeth and extending to the first and second molars. The flaps were lifted to or around the piriform aperture and separated from the nasal mucosa. The palatal mucoperiosteal flaps at the edge of the cleft were separated from the palate and lifted. After complete exposure of the cleft, the nasal mucosa at the base of the nostril was approximated and sutured. The palatal flaps were then inverted to create a soft tissue bundle. The defect was filled with cancellous bone from the ilium. To harvest this graft, first, a dermofat graft was harvested from the inguinal region as in the V-shaped mini flap method, and then a sufficient amount of cancellous bone graft was harvested by dissection. The grafted bone was compressed towards the cleft. Thus, osteoid material and cells per graft volume were maximized. The grafts were then covered with tension-free transposition using gingival mucoperiosteal flaps. The planned bone height was established as the height of the alveolar bone level of the adjacent tooth. After hemostasis control in the inguinal region and bone wax application to the donor area of the bone graft, subcutaneous and skin flaps were sutured. Cleft lip scar revision with a dermofat graft from the same area was repaired with the V-shaped mini flap method, and the operation was terminated.

### 2.4. Evaluation of cosmetic and functional outcomes

Cosmetic and functional outcomes were evaluated and recorded at 6 months after cleft lip scar revision using a V-shaped mini flap and dermofat graft combination. To avoid bias, the evaluations were performed by 2 surgeons who were not present during the patient’s surgery. A kappa analysis for reliability was performed between the 2 surgeons, and it was found to be 0.79, indicating a high level of inter-rater agreement. Lip closure, oral competence (lip tightness), lip symmetry, and labial contours were examined during whistling, speech, lip pursing, and articulation of bilabial sounds (/p/, /b/, /m/) in adult patients aged 16 years and older.^[16]^ Essentially, the desired outcomes were orbicularis oris muscle function, proper lip closure (oral competence), and smooth vermilion continence. In children under 16 years of age, improvement in lip tightness during sucking and feeding, the achievement of oral competence, and better continuity and symmetry at the vermilion border were examined.

### 2.5. Statistical analysis of data

After the data were coded by the researchers, statistical analysis of the data was performed using the Statistical Package for the Social Sciences 25.0 IBM (Armonk) statistical program. Descriptive statistical methods were used to analyze the data. Inter-rater agreement was calculated using SPSS-25 software (International Business Machines Corporation) – the kappa value.

### 2.6. Ethical aspects of the research

Before starting this study, Institutional Review Board approval was obtained from Dicle University Research and Application Hospital, Department of Plastic, Reconstructive, and Aesthetic Surgery. Then, the necessary ethical and legal permissions were obtained from the Dicle University Ethics Committee (Date: February 14, 2024, Decision No. 87) before starting the study. The Declaration of Helsinki was taken into consideration in the conduct of the study. Written and verbal informed consent was obtained from all patients whose data would be used.

## 3. Results

Table 1 shows the personal and clinical characteristics of the patients before cleft lip revision. The mean age of the patients was 15.30 ± 4.56 years, 53.3% of them were between 11 and 16 years old, 56.7% were female, 23.3% had whistling deformity, 26.7% had vermilion irregularity, 23.3% developed depression, 73.3% had pigmentation, and 56.7% had abnormal lip movements. Cleft lip was bilateral in 20% of the patients, and 40% had a history of concomitant cleft palate.

**Table 1:** Patients’ personal and clinical characteristics before cleft lip revision (n = 30).

Patient’ characteristics	Mean ± SD	Min–max
Age (mean ± SD)	15.30 ± 4.56	3–25

	n	%
Age		
10 yr and under	3	10
11–16	16	53.3
17–25	11	36.7
Gender		
Female	17	56.7
Male	13	43.3
Whistling deformity		
Yes	7	23.3
No	23	76.7
Vermillion irregularity		
Yes	8	26.7
No	22	73.3
Depression		
Yes	7	23.3
No	23	76.7
Pigmentation		
Yes	22	73.3
No	8	26.7
Abnormal lip movement		
Yes	17	56.7
No	13	43.3
Cleft lip type		
Unilateral	24	80
Bilateral	6	20
Concomitant cleft palate history		
Yes	12	40
No	18	60

SD = standard deviation.

Table 2 shows the flap and graft types used during cleft lip revision and the problems that developed after revision. During surgery, 60% of the patients underwent V-shaped mini flap, and 40% underwent V-shaped mini flap + Alveolar cleft repair. The primary cleft lip of 40% of the patients was complete. When the problems that developed after cleft lip revision were examined, hematoma developed in 6.7%, infection, wound dehiscence, and seroma in 10%, cyst formation in 6.7%, graft loss in 20%, and donor site morbidity in 13.4%. Ten percent of the patients underwent reoperation. Patients who experienced graft loss and underwent reoperation were treated with alveolar cleft repair with dermofat graft + bone graft. All patients had aesthetic deformity of the nose. One adult patient had a whistling deformity, and 1 pediatric patient had an articulation disorder at 6 months after cleft lip scar revision. The adult patient was referred to a physiotherapist, and the pediatric patient to a speech and language therapist.

**Table 2:** Characteristics associated with cleft lip revision and problems after revision (n = 30).

Patient’ characteristics	n	%
Cleft lip type		
Complete	12	40
Incomplete	18	60
Anesthesia type		
General	18	60
Local	12	40
Flap type		
V-shaped mini flap	18	60
V-shaped mini flap + Alveolar Cleft	12	40
Graft type		
Dermofat graft	18	60
Alveolar cleft repair with dermofat graft + bone graft	12	40
Receiving site issues/complications*		
Hematoma	2	6.7
Infection	3	10
Wound dehiscence (between fifth and seventh day)	3	10
Seroma	3	10
Cystification (third and sixth months)	2	6.7
Graft loss†	6	20
Reoperation†	3	10
Deformities in the nose	30	100
Donor site morbidity		
Hypertrophic scar development	2	6.7
Wound detachment	2	6.7
Cosmetic/functional outcomes (including those who underwent reoperation)		
Whistling deformity (adult)	1	3.3
Articulation disorder (child)	1	3.3

* More than 1 option for the same patient.

† Patients who experienced graft loss and underwent reoperation were treated with alveolar cleft repair using a dermofat graft + bone graft.

**Figure 1.**
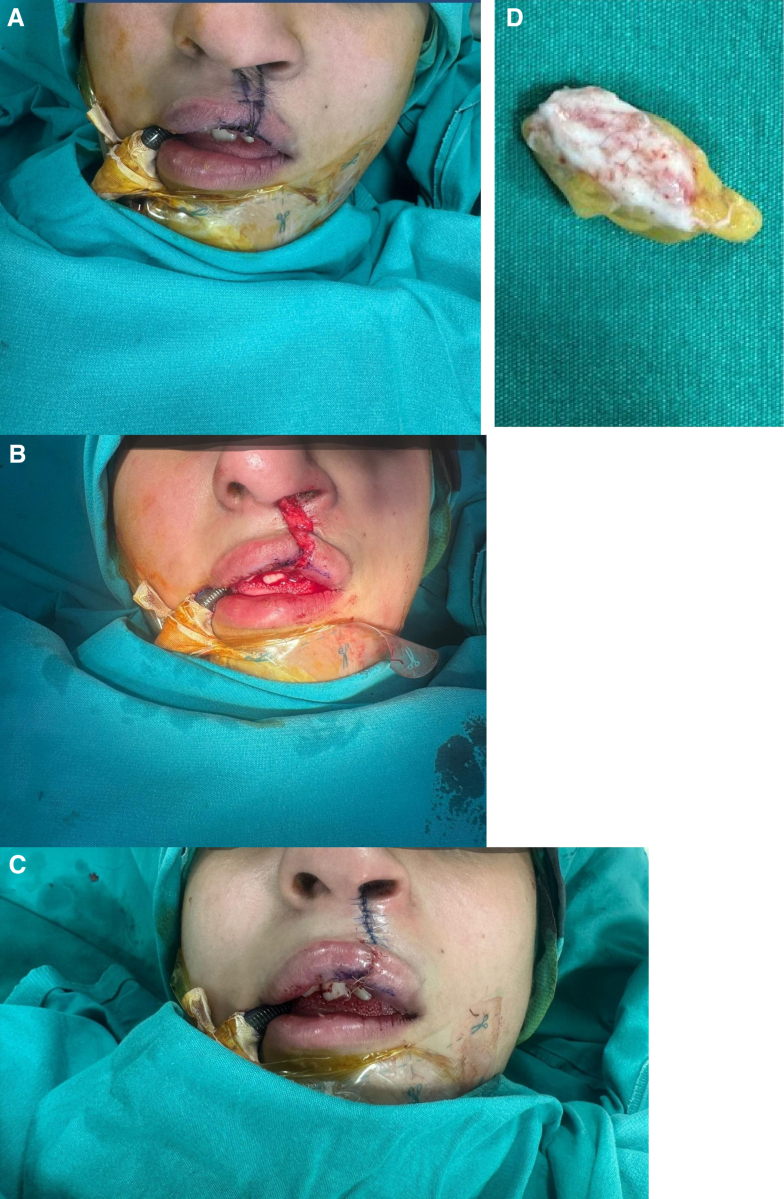
Use of V-shaped mini flap and dermofat graft in cleft lip revision. Seventeen-year-old female, previously operated with Millard technique for right complete cleft lip. (A) Scar tissue and flap marking, (B) excision of scar tissue and harvesting of the flap, (B, D) insertion of the dermofat graft, (C) suturing the wound lips by bringing them end to end at the marked points, (D) dermofat graft.

Figure 1 shows secondary cleft lip revision using a V-shaped mini flap and dermofat graft in cleft lip revision. A 17-year-old female patient underwent right complete cleft lip revision, and the primary repair was previously performed using the Millard technique. When the procedural steps of the secondary revision were examined, scar tissue and flap marking were performed first. Secondly, excision of the scar tissue and harvesting of the flap were performed (B). In the third stage, the dermofat graft was inserted (B, D), and in the fourth and final stage, the wound lips were brought end to end at the marked points and sutured (C).

**Figure 2.**
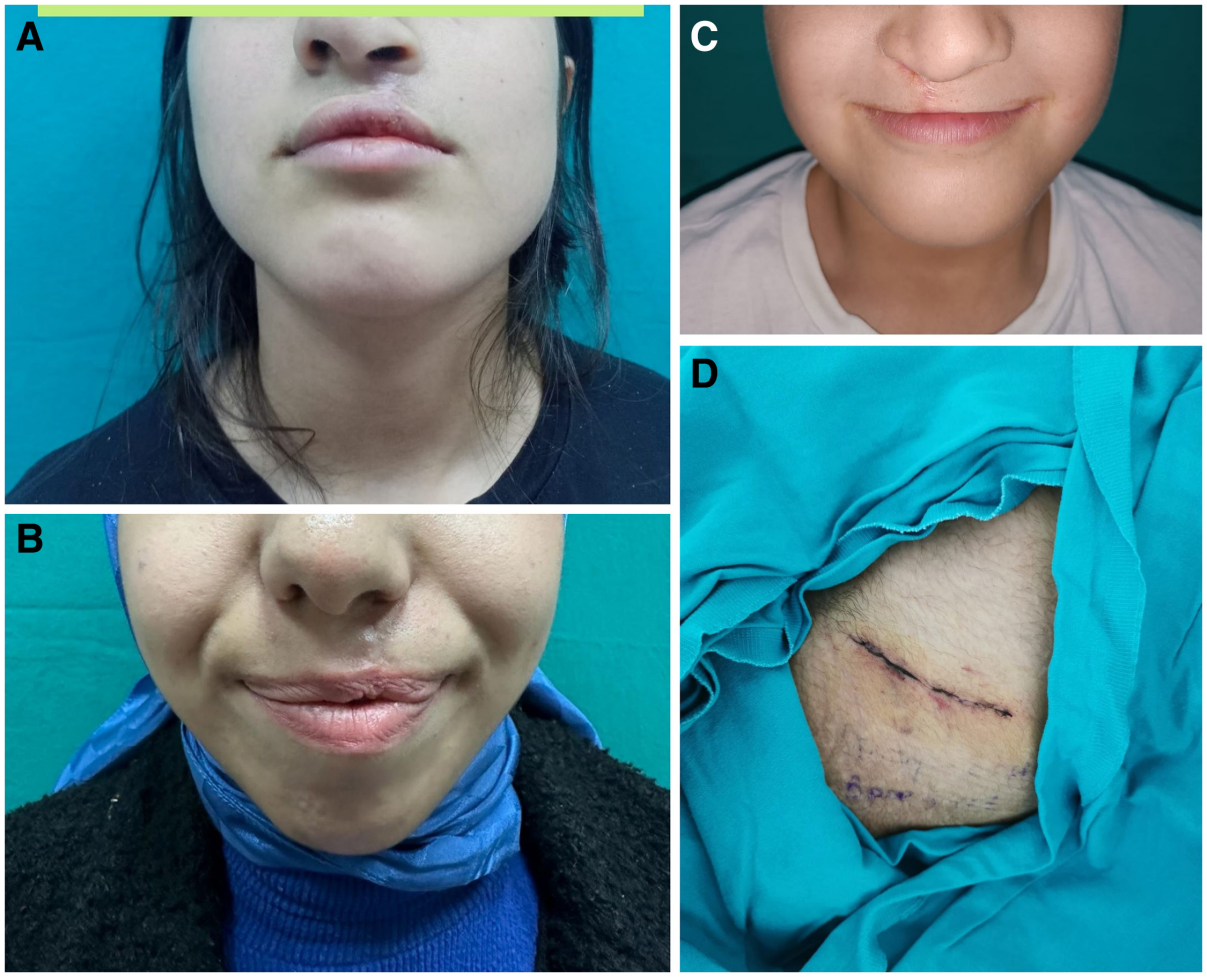
Recipient and donor area images after use of V-shaped mini flap and dermofat graft in cleft lip revision. (A) Thirteen years old female, left incomplete cleft lip previously operated with Millard technique, long term healing images taken from anterior and oblique angle after cleft lip revision (same patient), (B) 20 years old female, left incomplete cleft lip previously operated with Tenisson technique, long term healing images taken from anterior angle after cleft lip revision, (C) 13 years old male, previously operated with Tenisson technique for right complete cleft lip, long term healing images taken anteriorly after cleft lip revision, (D) donor site healed with minimal scarring.

Figure 2 shows the images of the recipient and donor sites after repair using a V-shaped mini flap and dermofat graft in cleft lip revision. According to (A), a 13-year-old female patient had a left complete cleft lip scar and had been previously operated on using the Millard technique. In (B), a 20-year-old female patient had a left incomplete (partial) cleft lip scar, and primary repair was performed using the Tenisson technique. In (C), a 13-year-old male patient had a right incomplete cleft lip scar, and primary repair was previously performed using the Tenisson technique. D showed a donor site healed with minimal scarring.

**Figure 3.**
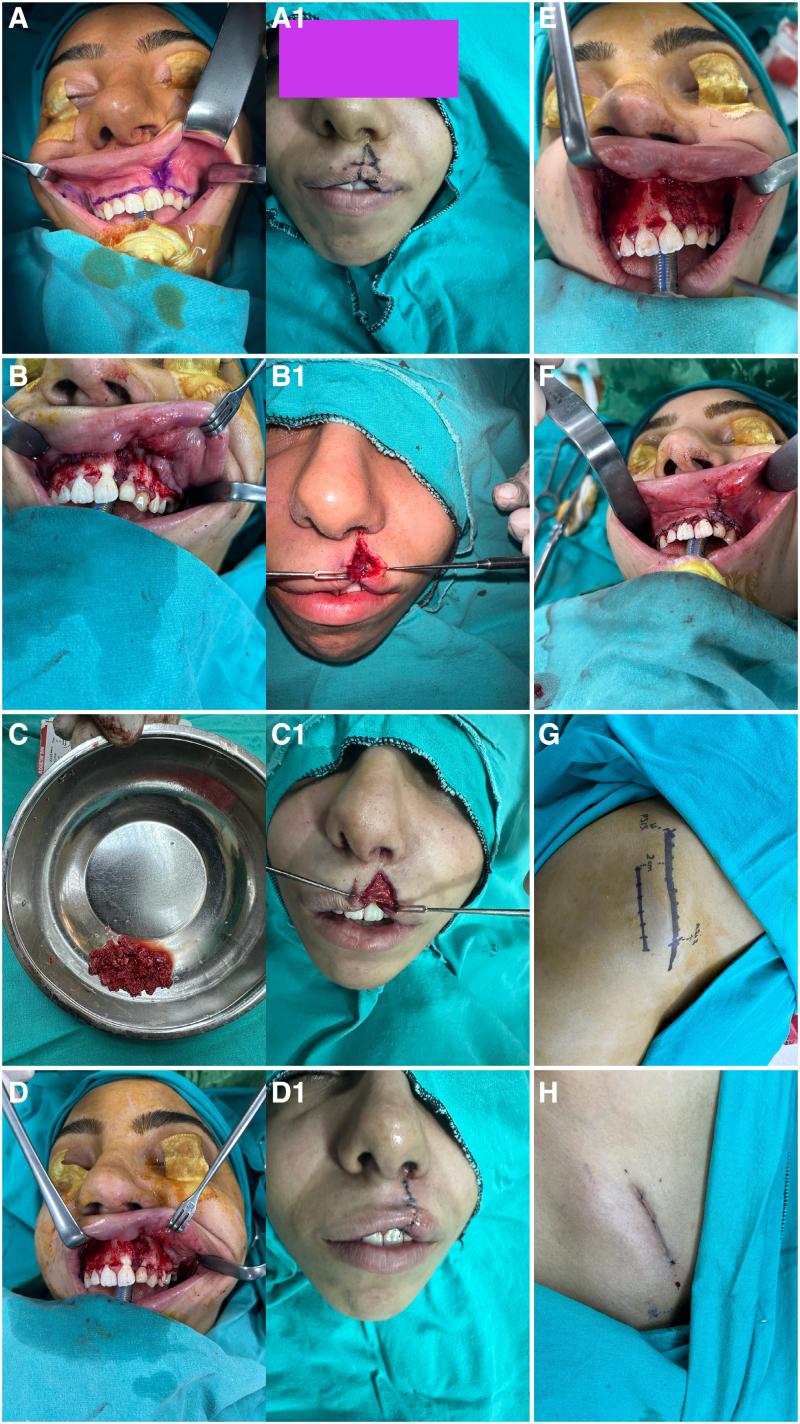
Alveolar cleft repair during cleft lip revision using V-shaped mini flap. A 20-year-old female, previously operated with the Tenisson technique for left incomplete (partial) cleft lip and alveolar cleft. (A) Gingival mucoperiosteal flaps with lateral and medial markings, (B) image of the bony cleft after elimination of the flaps, (C) image of the cancellous bone graft from the iliac crest, (D, E) image of the bone graft after application to the cleft area, (F) closure of the flaps without tension, (G) drawing of the rotor site for inguinal region, dermofat and cancellous bone graft, (H) closure of the donor area. (A1) Scar tissue and flap marking, (B1) excision of scar tissue and harvesting of flap, (C1) insertion of dermofat graft, (D1) suturing the wound lips by bringing them end to end at the marked points.

Figure 3 shows images of alveolar cleft repair during cleft lip revision using a V-shaped mini flap. In the first stage, gingival mucoperiosteal flaps were marked laterally and medially (A). In the second stage, the inguinal region, the dermofat, and the donor area for carnelian bone graft were marked (G); carnelian bone graft from the iliac crest (C) was applied to the cleft area (D, E), and the flaps were closed tension-free (F). Finally, the donor area was closed (H). In addition, the scar tissue and flap were marked in A1. In B1, the scar tissue was excised, and the flap was harvested. In C1, the dermofat graft was inserted, and in D1, the wound lips were sutured by bringing them end to end at the marked points.

**Figure 4.**
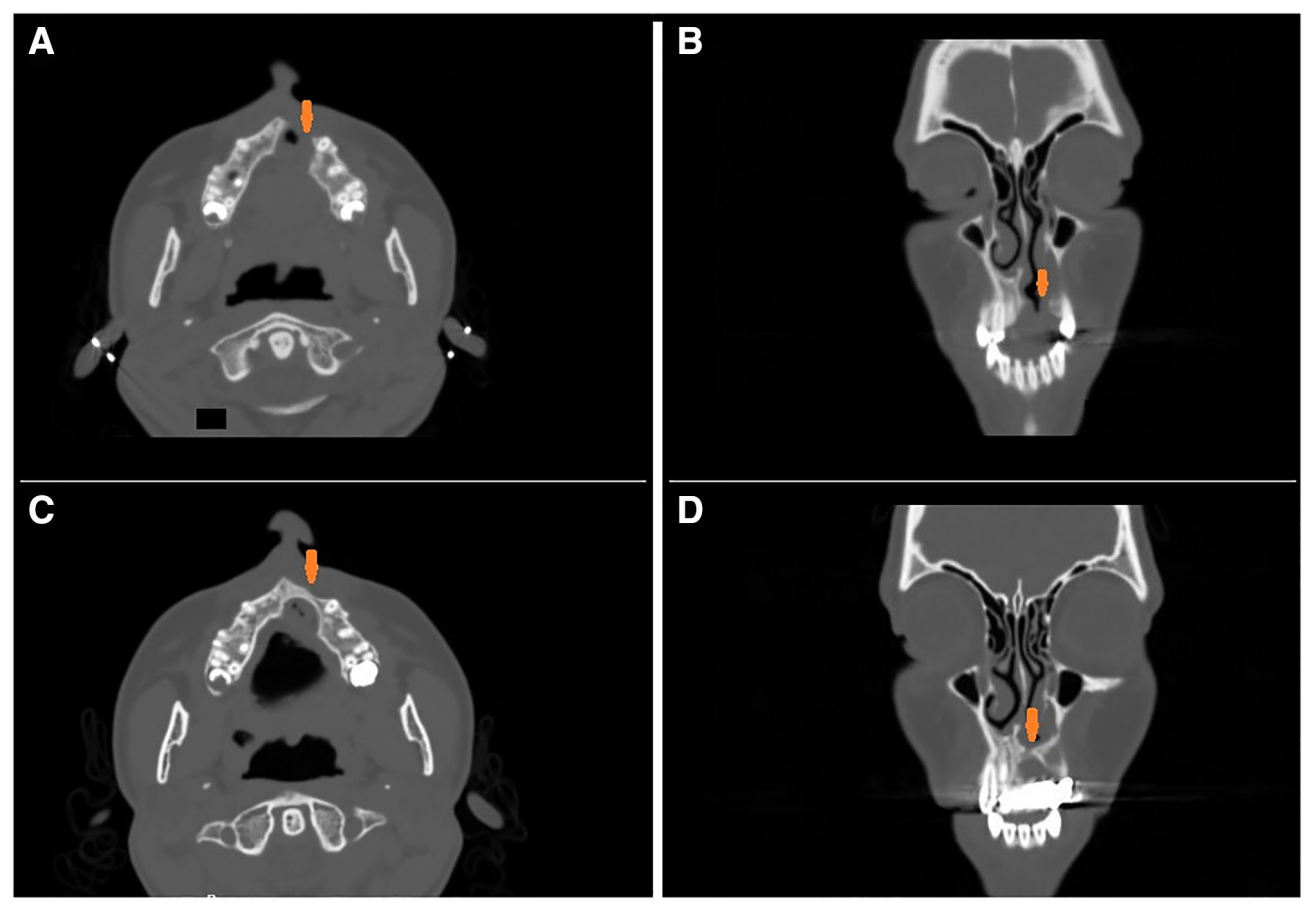
CT images of a patient with cleft lip accompanied by left alveolar cleft before secondary revision and postoperative 12th month. Figure 4 shows the CT scan result of a female patient (20 years old). (A, B) Preoperative tomography image of a patient with a left-sided alveolar cleft, (C, D) CT scan image at 12th month after alveolar cleft repair. CT = computed tomography.

Figure 4 shows the tomography images of a patient with a cleft lip accompanied by a left alveolar cleft before secondary revision and at the 12th postoperative month. The need for an alveolar cleft operation is seen in this patient who was planned for a secondary revision operation for the cleft lip scar.

## 4. Discussion

Cleft lip scars often manifest as asymmetry and irregular lip contour, and a weak free lip edge reveals the need for revision. After the initial repair of a severe cleft, the possibility of secondary revision is anticipated. In this study, a V-shaped mini-flap was positioned at the very tip of the vermilion border and cut along the vermilion border, thus realizing the revision of the vermilion border notch. The main aim of secondary revision of cleft lip is to increase functionality, eliminate deformity and asymmetry, and improve the patient psychosocially.^[17]^ Ten percent of patients with complete cleft lip required a third revision. Although the timing of secondary revision has not yet been clarified,^[18]^ the incidence rate is as high as 56.9%.^[19,20]^ Another study reported a 3:1 ratio of patients requiring free margin reoperation after unilateral complete cleft repair.^[21]^ For our study sample, the aforementioned ratio was approximately 2:1, while the rate of a third operation after secondary revision was 5:1. The main indications for secondary revision are hypertrophic and/or enlarged scars, asymmetrical lip, Cupid’s bow and whistle deformity, vermilion irregularity, and muscle deformities.^[22-24]^ The reasons for secondary revision of cleft lip in this study were consistent with the literature.

The superficial deformity involving the skin, subcutaneous tissue, and mucosa^[25]^ is often revised only for aesthetic concerns and requires less laborious procedures (injections and laser therapies, etc), and many techniques can be used in its solution. In cleft lip revisions accompanied by muscle deformities, secondary revision is performed using the primary reconstruction technique after the defect is reconstructed, which is called complete revision.^[26]^ Incomplete cleft lip is partial, whereas complete cleft lip extends to the base of the nose. The need for secondary revision after the primary repair of cleft lip, regardless of the age of the patient, can be considered a complication of the first operation. Plastic surgeons focus on optimal healing in meeting this need, which often starts at an early age. The first consideration is staging using an effective grading scale to determine the severity of the lip defect.^[23]^ Once the severity of the defect is determined, noninvasive/minimally invasive, or invasive options (up to full revision cheiloplasty) can be reviewed, and the treatment method with the least morbidity can be selected, respectively.^[8,26-28]^ In this study, the positioning of this V-shaped mini flap can prevent vermilion deviation caused by scar contracture that may occur postoperatively. The dermofat graft from the inguinal region was placed in the recipient site. After suturing, the orbicularis oris muscle was revised by giving an overlap shape.

In this study, patients who experienced graft loss after secondary revision and underwent reoperation had a complete cleft lip type, and dermofat graft + bone graft was used in alveolar cleft repair. It is not surprising that this group of patients experienced problems in postoperative healing. In the Southeastern Anatolia region where the study was conducted, socio-cultural and economic deficiencies lead to problems such as hygiene issues and a lack of understanding or application of postoperative education. This is related to the failure to protect and keep the surgical wound clean. All these factors may have triggered the development of postoperative complications. In our study, the rate of patients requiring tertiary revision after secondary revision was 3 out of 30, and no problems developed after the third revision. Apart from these 3 patients, 1 adult with whistling deformity and 1 child with articulation disorder were reported. Whistle deformity after primary cleft lip repair is characterized by fibrosis resulting from failure to regulate muscle tension during surgery, disrupting soft tissue function and causing lip deformity.^[29]^ Dermofat graft is advantageous in terms of minimal donor site morbidity.^[30]^ Our results are consistent with previous literature.

In patients who were to undergo alveolar bone grafting with cleft lip scar revision, alveolar cleft repair and then cleft lip scar revision were performed by the senior author (MFA). The grafted bone was compressed towards the cleft. Thus, osteoid material and cells per graft volume were maximized, and then the grafts were covered with tension-free transposition using gingival mucoperiosteal flaps. Bone height was realized as the height of the alveolar bone level of the adjacent tooth. In this study, ten percent of patients with complete cleft lip required a third revision. These patients had undergone secondary revision with alveolar cleft repair using dermofat graft plus bone graft. During the revision of cleft lip, alveolar bone grafting is performed to provide bone support for teeth located very close to the cleft region, fixation of the maxillary arch, orthodontic treatment, and repair of oronasal fistula.^[31]^ In our study, the indication for alveolar bone grafting was the presence of an alveolar cleft in addition to cleft lip scar revision. Alveolar bone grafting is performed for permanent alignment of teeth, periodontal support, and alveolar cleft reconstruction.^[32]^ A previous study recommended osteotomy during grafting to improve maxillary hypoplasia. However, the same study associated osteotomy with shallowing of the labia, low alveolar bone level, and surgical recurrence.^[33]^ This shows that the safety of maxillary osteotomy is controversial. Maxillary osteotomy was not performed in this study.

Later septoplasty may be required for low nasal dorsum, nasal asymmetry, and deprojected type after cleft lip deformity.^[34]^ Caudal septum deviation and inferior turbinate hypertrophy may cause nasal airway obstruction after cleft lip repair.^[35]^ Reduction of the turbinate and septum repair is an ideal surgical treatment option.^[36]^ Indeed, all patients who underwent revision of cleft lip scars in this study had deformities in the nose. Performing septorhinoplasty in these patients at a later stage appears to be a beneficial solution. This study had several limitations. The retrospective nature of the study is not ideal for reporting and evaluating the superiority of a surgical technique. The study lacks objective outcome measurement, blinding of assessors to the technique, comparison of the current technique with others, patient satisfaction and patient-reported outcomes, and a standardized photographic protocol to document lip function assessment. A heterogeneous patient population of 3 to 25 years leads to different healing and scarring in the tissue. Single-surgeon outcomes, the small sample size, and the fact that it was a single-center study limit generalizability. Although at a low level, wound site and donor site complications developed, which limit the use of the V-shaped mini flap method and dermofat graft together or the use of secondary revision when alveolar cleft repair is included in the same session.

## 5. Conclusion

Secondary revision with V-shaped mini-flap or V-shaped mini-flap + alveolar bone graft was performed to reconstruct the cosmetic and functional defects that arise after primary cleft lip repair in this study. This surgical technique can be added to previous techniques because it has advantages. Reoperation and graft loss were major complications in a small number of patients; other complications were few and minor.

## Author contributions

**Conceptualization:** Mehmet Fatih Akkoc, Volkan Ozel, Mehmet Bayram, Semra Bulbuloglu, Ibrahim Ugur Budak, Berk Tahhan, Ali Haydar Cinbas, Ismail Yildiz.

**Data curation:** Mehmet Fatih Akkoc, Volkan Ozel, Mehmet Bayram, Semra Bulbuloglu, Ibrahim Ugur Budak, Berk Tahhan, Ali Haydar Cinbas, Ismail Yildiz.

**Formal analysis:** Mehmet Fatih Akkoc, Volkan Ozel, Mehmet Bayram, Semra Bulbuloglu, Ibrahim Ugur Budak, Berk Tahhan, Ali Haydar Cinbas, Ismail Yildiz.

**Funding acquisition:** Mehmet Fatih Akkoc, Volkan Ozel, Mehmet Bayram, Semra Bulbuloglu, Ibrahim Ugur Budak, Berk Tahhan, Ali Haydar Cinbas, Ismail Yildiz.

**Investigation:** Mehmet Fatih Akkoc, Volkan Ozel, Mehmet Bayram, Semra Bulbuloglu, Ibrahim Ugur Budak, Berk Tahhan, Ali Haydar Cinbas, Ismail Yildiz.

**Methodology:** Mehmet Fatih Akkoc, Volkan Ozel, Mehmet Bayram, Semra Bulbuloglu, Ibrahim Ugur Budak, Berk Tahhan, Ali Haydar Cinbas, Ismail Yildiz.

**Project administration:** Mehmet Fatih Akkoc, Volkan Ozel, Mehmet Bayram, Semra Bulbuloglu, Ibrahim Ugur Budak, Berk Tahhan, Ali Haydar Cinbas, Ismail Yildiz.

**Resources:** Mehmet Fatih Akkoc, Volkan Ozel, Mehmet Bayram, Semra Bulbuloglu, Ibrahim Ugur Budak, Berk Tahhan, Ali Haydar Cinbas, Ismail Yildiz.

**Software:** Mehmet Fatih Akkoc, Volkan Ozel, Mehmet Bayram, Semra Bulbuloglu, Ibrahim Ugur Budak, Berk Tahhan, Ali Haydar Cinbas, Ismail Yildiz.

**Supervision:** Mehmet Fatih Akkoc, Volkan Ozel, Mehmet Bayram, Semra Bulbuloglu, Ibrahim Ugur Budak, Berk Tahhan, Ali Haydar Cinbas, Ismail Yildiz.

**Validation:** Mehmet Fatih Akkoc, Volkan Ozel, Mehmet Bayram, Semra Bulbuloglu, Ibrahim Ugur Budak, Berk Tahhan, Ali Haydar Cinbas, Ismail Yildiz.

**Visualization:** Mehmet Fatih Akkoc, Volkan Ozel, Mehmet Bayram, Semra Bulbuloglu, Ibrahim Ugur Budak, Berk Tahhan, Ali Haydar Cinbas, Ismail Yildiz.

**Writing – original draft:** Mehmet Fatih Akkoc, Volkan Ozel, Mehmet Bayram, Semra Bulbuloglu, Ibrahim Ugur Budak, Berk Tahhan, Ali Haydar Cinbas, Ismail Yildiz.

**Writing – review & editing:** Mehmet Fatih Akkoc, Volkan Ozel, Mehmet Bayram, Semra Bulbuloglu, Ibrahim Ugur Budak, Berk Tahhan, Ali Haydar Cinbas, Ismail Yildiz.
